# Advances in the Removal of Cr(III) from Spent Industrial Effluents—A Review

**DOI:** 10.3390/ma16010378

**Published:** 2022-12-30

**Authors:** Katarzyna Staszak, Izabela Kruszelnicka, Dobrochna Ginter-Kramarczyk, Wojciech Góra, Marek Baraniak, Grzegorz Lota, Magdalena Regel-Rosocka

**Affiliations:** 1 Institute of Chemical Technology and Engineering, Faculty of Chemical Technology, Poznan University of Technology, ul. Berdychowo 4, 60-965 Poznan, Poland; 2 Department of Water Supply and Bioeconomy, Faculty of Environmental Engineering and Energy, Poznan University of Technology, ul. Berdychowo 4, 60-965 Poznan, Poland; 3 Institute of Chemistry and Technical Electrochemistry, Faculty of Chemical Technology, Poznan University of Technology, ul. Berdychowo 4, 60-965 Poznan, Poland

**Keywords:** chromium(III) removal, industrial effluents, adsorption, precipitation, liquid–liquid extraction, membrane techniques, electrochemical techniques, low environmental impact technologies

## Abstract

The review presents advances in the removal of Cr(III) from the industrial effluents published in the last ten years. Although Cr(III) has low solubility and is less dangerous for the aquatic environment than Cr(VI), it cannot be released into the aquatic environment without limitations and its content in water should be restricted. The development of efficient techniques for the removal of Cr(III) is also a response to the problem of chromium wastewater containing Cr(VI) ions. Very often the first step in dealing with such wastewater is the reduction in chromium content. In some cases, removal of Cr(III) from wastewaters is an important step for pretreatment of solutions to prepare them for subsequent recovery of other metals. In the review, hydrometallurgical operations for Cr(III) removal are presented, including examples of Cr(III) recovery from real industrial effluents with precipitation, adsorption, ion exchange, extraction, membrane techniques, microbial-enhanced techniques, electrochemical methods. The advantages and disadvantages of the operations mentioned are also presented. Finally, perspectives for the future in line with circular economy and low-environmental impact are briefly discussed.

## 1. Introduction

The issue of minimizing waste generation and reducing its harmful potential for the environment has become a permanent feature of new industrial solutions. Research focused on this aspect shows a wide range of possibilities, including the choice of techniques that reduce waste generation (e.g., process optimization) or techniques that enable waste sources to be treated, with a consequent reduction in emissions. Although it is, of course, far better to implement the ‘prevention is better than cure’ procedure, this is not always an option. Therefore, the development of waste source treatment techniques should be prioritized.

**Figure 1:**
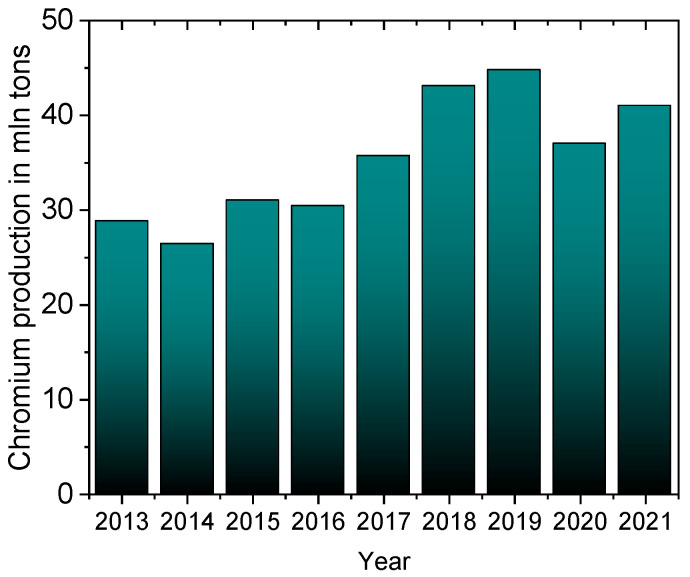
Chromium mine production worldwide from 2010 to 2021 [2].

One serious environmental problem is the release of heavy metals, including chromium, into the environment. Chromium occurs in nature in the form of minerals such as crocoite (PbCrO_4_), chromite (FeCr_2_O_4_), or lopesite (Na_2_Cr_2_O_7_). This metal is applied to harden steel and protect it from corrosion, which is why it is a component of stainless steels and different alloys, from which special and everyday products are made, such as airplanes, tanks, machinery, industrial installations, kitchen utensils, surgical tools, etc. [1]. As chromium forms a passivation layer, a fine and solid chromium oxide film, it is used as an external coating to protect the internal metal (steel) from corrosion. Chrome plating is widely used in the leather tanning industry and in the manufacturing of steel products, which has been accelerating the consumption of chromium worldwide for the last 10 years (Figure 1) [2]. In 2021, the global chrome plating market was estimated at $16.6 billion, and is expected to grow to $22.2 billion by 2028 [3].

Chromium compounds are used in many chemical processes as industrial catalysts and pigments for glass, porcelain glazes (bright green, yellow, red, and orange). Approximately 90% of all leather is tanned with chrome [4,5], and toxic waste tannery effluents are generated and must be efficiently purified before release.

In the aquatic environment, chromium is found naturally in rainwater (0.2–1 µg/dm^3^), seawater (0.04–0.5 µg/dm^3^), surface waters (0.5–2 µg/dm^3^) and groundwater (<1 µg/dm^3^) [6]. Natural water can be contaminated by anthropogenic chromium, especially from tanneries. Chromium is present in aqueous solutions in different forms, trivalent and hexavalent being the prevalent ones. Cr(III) has low solubility and is less dangerous to the aquatic environment than Cr(VI), which shows much better solubility and can easily migrate through the groundwater, mix with it, and contaminate it [7]. The harmfulness of chromium is well known [8,9,10]: toxicity limits are 28–80 mg/dm^3^ for fish, 0.05 mg/dm^3^ for drinking water. However, mutations induced by the chromium(III) complex with 2,2′-bipyridyl were found, among others, in oxidation-sensitive *Salmonella* strains TA2638 and TA102 [11].

Although Cr(III) is less toxic to living organisms (negative results in most mutagenicity tests) than Cr(VI) and trace quantities of Cr(III) are even essential for proper functioning of the human organism, discharge of Cr(III) present in large quantities in spent tanning liquors is burdensome for the environment. For example, groundwater contamination with chromium near tanneries around the world must be carefully monitored [7,12,13]. An additional risk is the fact that Cr(III) can be oxidized to hexavalent chromium in natural water and soil [13].

There are no unified discharge limits for Cr(III) or Cr(VI) to the aquatic environment not only in different parts of the world but also within the EU or the World Health Organization. Each country establishes its own standards for the chromium discharge limits to various aquatic systems (marine water, lake, river, and sewer system). The maximum discharge limit to the aquatic environment in the EU is 0.05–2 and 5 mg/dm^3^ for Cr(VI) and Cr_total_, respectively [14,15].

It should be noted that, depending on the industry in which wastewater is generated, the presence of associated components, for example, surfactants, other metal ions, acids, and bases must be taken into account. Therefore, it is not possible to unequivocally identify one single best method for chromium removal. Moreover, as science progresses, new materials, techniques, and solutions can be expected to solve the problem of chromium separation from aqueous systems.

Taking into account the existing problem of chromium in wastewater and the development of separation techniques, the aim of this review is to present the latest trends in the management of real industrial effluents containing Cr(III). In addition, the challenges and perspectives facing researchers in this area were analyzed.

## 2. Origin and Composition of Spent Industrial Effluents

Chromium-containing waste effluents can originate not only from steel plating and tannery processes, but also can be generated as a result of the leaching of various types of steel [16,17,18,19]. The removal of chromium from industrial effluents is important not only for the removal of hazardous metal ions but also for the purification of the effluents before further steps of treatment/recovery of valuable metals.

In order to provide an initial indication of the complexity of the chromium effluent problem, the exemplary compositions of Cr(III)-containing industrial effluents are summarized in Table 1.

Industrial effluents contain mostly Cr(VI); thus, the research efforts are focused on removal of toxic hexavalent species. However, Cr(III) is present in passivation baths or leach solutions. As can be seen in the summary presented, it is important to keep in mind that the parameters such as concentration and pH will influence the choice of the treatment method [5]. In general, three methods are possible to reduce Cr(III) concentrations from industrial effluents. These are the following: (i) oxidation to Cr(VI) compounds, (ii) reduction to the metallic element, or (iii) co-precipitation with ions of other low-toxic metals without changing the oxidation state. The former option, due to high toxicity of Cr(VI) relative to Cr(III), is not very promising, since nowadays many technologies are abandoning the use of Cr(VI) compounds.

## 3. Hydrometallurgical Methods for the Removal of Cr(III) from Waste Effluents

### 3.1. Precipitation

Chemical precipitation is one of the most widely used methods for the removal of heavy metals from inorganic effluents in industry due to simple operation [35,36]. Conventional chemical precipitation processes produce insoluble precipitates of heavy metals such as hydroxide, sulfide, carbonate, and phosphate. The mechanism of precipitation is based on the production of an insoluble metal precipitation by reacting the metals dissolved in the solution and a precipitant. As a result of precipitation, fine particles are generated and chemical precipitants, coagulants, and flocculants are used to increase the size of precipitated particles and to facilitate their removal as a sludge. Once the metals precipitate and form solids, they can be easily removed, and effluents of low metal concentrations can be discharged. The removal percentage of metal ions in the solution may be improved to optimum by changing major parameters such as pH, temperature, initial concentration, and/or charge of ions, etc. [37]. Hydroxide and sulfide precipitation [38,39] are the most commonly used and efficient methods for the removal of heavy metal ions from industrial effluents [40]. However, each of these precipitation methods has its own limitations in the treatment of effluents of mixed heavy metals. For hydroxide precipitation, all metal hydroxides do not completely precipitate at the same pH because each type of metal hydroxide is favored by precipitation in a certain pH range [38]. According to Ye et al. [41], the precipitation operation also produces large volumes of relatively low density sludge that need further treatment before disposal [36]. This method of removal of metal ions is widely used because of its relative simplicity, easy control of pH, and low cost. Sulfide precipitation also constitutes an effective process for treating toxic heavy metal ions. The advantage of sulfide precipitation compared to hydroxide precipitation is that there is a much lower residual concentration of the metal ions in the effluents. The solubility of the metal sulfide deposits is substantially lower than that of the hydroxides, and the sulfides are not amphoteric. In addition, the sulfide precipitates also exhibit better thickening and can achieve a high degree of metal removal in a wide pH range compared to the hydroxide precipitation. However, sulfide precipitation is costly and can emit hydrogen sulfide gas (H_2_S) under acidic conditions [39,42].

For example, Uddin et al. [42] have explored the potential of using NaHCO_3_ and CaCO_3_ compared to MgO, as precipitating agents for the removal and the recovery of chromium from chrome tannery wastewater. For NaHCO_3_ and CaCO_3_, the maximum removal efficiency of Cr(III), 99.97 and 99.95%, respectively, was found at pH 8.31 and 8.9, while MgO showed the highest efficiency (99.98%) at pH 8.91. The highest efficiency of the three precipitating agents was observed at a dose of 0.5 g/250 cm^3^ (2000 mg/cm^3^). The advantage of these precipitants (i.e., NaHCO_3_, CaCO_3_, and MgO) is their high availability and low cost compared to other precipitating agents. It should be emphasized that the environmental impact of chromium hydroxide landfill can be minimized by recovering Cr(III) from the sludge and reuse of chromium in the tanning process.

Kostrzewa et al. [17] carried out Cr(III) precipitation with solutions of 3 or 30% NaOH, 10% CaO, 10% NaHCO_3_ and 10% Na_2_CO_3_ from real sulfate solutions after steel leaching (PLS); for the PLS composition, see Table 1. As expected, with increasing pH (pH values 3, 4, 5), the yield of precipitation increased. It was revealed that almost the entire amount of Cr(III) was precipitated and separated as a solid from the PLS. The precipitation yield exceeded 99% for the three reagents NaOH, CaO, and NaHCO_3_ at pH 5. It should be noted that precipitation with the use of NaOH and CaO solutions led to a complete removal of Cr(III) at both pH 4 and 5. However, it should be emphasized that precipitation was not selective: quantitative co-precipitation of Al(OH)_3_ and partial formation of deposit of Ni(OH)_2_ and Co(OH)_2_ were reported. Despite some problems with selectivity, out of the four reagents investigated, the NaOH solution was recommended as the best precipitating agent for Cr(III) removal from the sulfate solutions. In addition, Kokkinos and Zouboulis [43] confirmed the high efficiency of NaOH and Ca(OH)_2_ in the removal of Cr(III) from the leachate/filtrate containing 5.2 g/dm^3^ Cr(III). The precipitation efficiency exceeded 99% for pH values above 7, and no significant differences between the effectiveness of these two reagents were observed.

The optimum pH values for Cr(OH)_3_ precipitation with various precipitants are presented in Table 2.

To sum up, despite the low cost and simplicity of the precipitation operation, other methods for the removal of metal ions from industrial effluents have been developed focusing on the selectivity of the separation of various metal ions and the low environmental impact of the method.

### 3.2. Adsorption

Among the methods of metal ion removal, the adsorption technique has recently gained prominence due to its flexibility in operation, process design, and significant effect on reduction in toxicity and biological availability of heavy metals in wastewaters [44]. The three main steps of adsorption onto solid sorbent involve the transport of the contaminant to the sorbent surface from the aqueous solution, adsorption onto the solid surface, and transport within the sorbent particle. The charged contaminants tend to adsorb on oppositely charged adsorbents via electrostatic force of attraction. Heavy metals show a strong affinity for surface hydroxyl or other functional groups. Since adsorption is often reversible, it is accompanied by the desorption step (a reverse process to adsorption, in which the adsorbate ions are transferred from the adsorbent surface to an eluting solution), and the adsorbents can be regenerated for multiple uses, making the adsorption a cost-effective and high-efficiency process to produce high quality treated effluents. Langmuir maximum adsorption capacity (q_max_) and Freundlich adsorption isotherm (K_F_) are parameters that are generally used to interpret metal adsorption on various materials [45,46]. There are several factors that influence the adsorption efficiency of adsorbents, including temperature, pH, stirring duration, adsorbent-to-effluent ratio, and the initial concentration of metal ions. The efficiency of heavy metal adsorption typically increases with the rise of the factors mentioned in Table 3 [45].

Activated carbon is one of the most studied adsorbents. Many researchers have established the efficiency of activated carbon and activated carbon composites as sorbents in the removal of many types of pollutants including heavy metals and dyes. The adsorption mechanism has been reported to occur in four consecutive steps: (1) bulk transport (the heavy metal ions transport in the solution phase); (2) film transport (the heavy metal ions are transported from the bulk liquid phase to the external surface of the adsorbent through a hydrodynamic boundary layer or film); (3) intraparticle (diffusion of the heavy metal ions from the exterior of the adsorbent into the pores of the adsorbent); and (4) adsorption. Peng and Guo [35] studies have shown that melamine achieved high adsorption capacity (2843 mg/g) and removal efficiency of Cr(III) (98.63%) in 60 min at n_(melamine)_/n_(Cr)_ = 1.5 and a reaction temperature of 90 °C. The adsorption isotherm and kinetic model indicated that the predominant mechanisms of Cr(III) adsorption are the electrostatic attraction and stacking interaction. It has been also reported that the Cr(III) adsorption was a spontaneous, endothermic and physisorption process.

**Figure 2:**
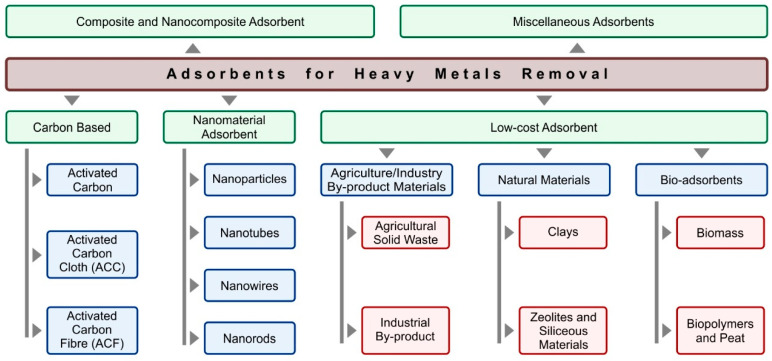
The classification of adsorbents used to remove heavy metals [47,48].

In recent years, biosorption as an ecofriendly technique for Cr(III) removal has attracted much attention. The major advantages of biosorption over conventional adsorption are low cost, high adsorption capacity, and good selectivity. The classification of adsorbents used for the removal of heavy metal ions is shown in Figure 2.

The adsorption capacity for the removal of chromium by various biosorbents is presented in Table 4. The biosorption intensity of raw and chemically modified sawdust (SD) and corn husk (CH) has been investigated by Afzaal et al. [49] to eliminate Cr(III) from the aqueous solutions. SD and CH were used as biosorbents and chemically treated with H_2_SO_4_, NaOH, and detergent powder. The biosorption potential was estimated on the basis of the percentage removal efficiency (RE) of Cr(III) and the adsorption capacity (q_max_). The detergent-treated SD (DTSD) and detergent-treated CH (DTCH) were reported to show the highest Cr(III) removal of 9.27 ± 0.15% and 99.16 ± 0.08% RE, respectively. Similarly, base-treated SD (BTSD) and base-treated CH (BTCH) exhibited 95.53 ± 0.18% and 92.43 ± 0.22% RE compared to 77.87 ± 1.64% and 81.96 ± 0.34% RE with acid-treated SD (ATSD) and acid-treated CH (ATCH), respectively. Raw SD (RSD) and raw CH (RCH) showed lower Cr(III) removal, i.e., 23.68 ± 1.52% and 35.52 ± 4.74%.

Alkaline-treated peel (with 0.005–0.15 M NaOH solution) of *Artocarpus nobilis* fruit was applied as a biosorbent for the removal of Cr(III) and Cr(VI) species from aqueous solutions [50]. A series of experiments performed within a wide range of solution pH demonstrated that the optimum pH for Cr(III) removal was pH 5.0. The highest Cr(III) adsorption of 4.89 × 10^3^ mg/kg (pH = 5) was achieved on the biosorbent treated with a solution of 0.010 M NaOH, and the pseudo-first-order kinetics were reported at the ambient temperature of 27.5 °C and were unchanged when the solution temperature was increased up to 40.0 °C. Furthermore, the activation energy for adsorption of Cr(III) was determined to be 66.82 kJ/mol, suggesting a strong attraction between the adsorbate and the biosorbent.

**Table 4:** Comparison of adsorption capacities of Cr(III) on various adsorbents [51].

Materials	Adsorption Capacity (mg/g)
Rice husk	0.79
Raw rice bran	0.8
Coconut shell charcoal	3.65
Modified rice hull	23.4
Activated alumina	1.6
Activated charcoal	0.9614
Wheat bran	0.942
Activated rice husk carbon	0.8
Pine leaves	0.277
Modified oak sawdust	1.7
CETYL-amended zeolite	0.65
Cornelian cherry	59.4
Apricot stone	59.64
Sodium carboxy methyl cellulose stabilized iron nanoparticles	255.0
Scrap iron	19.0
Fe@SiO_2_	467.0
Wool	41.2
Olive cake	33.4
Magnetic calcite	24.2

The dried microbial biomass (particle size of 100–150 μm) produced from four microbial strains isolated from leather factory sewage sludge was investigated as a low concentration Cr(III) biosorbent by four microbial powder biosorbents (*Trichosporonales* sp. (TP), *Bacillus cereus* (XB, MY) and *Aspergillus terreus* (TQ)) [52]. All biosorbents showed promising sorption efficiency towards Cr(III), and the groups of hydroxyl, carboxyl, polyglucose, amine, phosphoric acid, and sulfur functional groups played an important role in Cr(III) adsorption. The equilibrium adsorption capacity decreased in the following order: XB > MY > TP > TQ (11.51, 10.5, 7.75 and 9.85 mg Cr(III)/g, respectively). Based on the analysis of isotherm models (Langmuir, Freundlich, Temkin, and Dubinin-Radushkevich isotherm models), the biosorption process for all of the studied adsorbents was reported to follow three phases: rapid phase, slow phase, and equilibrium. The first phase was the rate-controlling one. Furthermore, the four biosorbents were regenerated by desorption and still had adsorption performance after four times of reuse. It was proven that the four biosorbents could be feasible biomass for the removal of low concentration Cr(III) from aqueous solutions.

The lignocellulosic biomass of *Citrus limon* peel (CLP) powder was proposed for an ecofriendly biosorption/bioremoval process of Cr(III), Cu(II) and Pb(II) ions from an aqueous solution [53]. Under optimal conditions, the maximum removal of Cr(III), Cu(II) and Pb(II) ions was 97.47, 87.13 and 95.71%, respectively. The presented bioremoval processes by prepared CLP biosorbent were proven to be temperature-independent. The Langmuir isotherm model was found to be an excellent fit of the isotherm data with the calculated biosorption capacities of 111.11 mg Cr(III)/g.

Among many other biosorbent materials, keratinous materials contain intricate networks of stable and water-insoluble fibers with large surface areas and are abundant biogenic bioresources [54,55,56]. In particular, human hair composed of keratin is considered to be a widespread waste, and its accumulation can cause environmental problems. Similarly to microbial biomass, this biomaterial is also abundant in carboxyl, amido, and disulfide groups and can serve as a good biosorbent of heavy metals such as Cr(III), Mn(II), Co(II), Ni(II), Cu(II), Zn(II), Cd(II) and Pb(II) from multiple aqueous solutions. Considering the important role of the chemical structure and surface properties of human hair in the metal biosorption process, two different kinds of hair, i.e., untreated human hair (H1) and periodically bleached and dyed hair (H2) were tested for the biosorption effectiveness of heavy metal ions. Owing to the active functional groups (especially sulfonate) formed during bleaching and dyeing treatments and the larger relative surface area, the treated hair (H2) showed better biosorption capacity than the untreated H1. The biosorption capacities of the heavy metal ions followed the order Cu(II) > Pb(II) > Cr(III) > Zn(II) > Cd(II) > Ni(II) > Co(II) > Mn(II) for H2, and Cu(II) > Cr(III) > Pb(II) for H1; the other heavy metal ions were not adsorbed by H1 at pH 4.0. H2 showed a faster biosorption rate than H1. For the multiple heavy metal solution, the maximum removal efficiencies of Cu(II), Cr(III) and Pb(II) were higher than 90 and 95% for H1 and H2, respectively. Thus, human hair can be pointed out as a potential biosorbent for the removal of heavy metal ions.

In addition, a polymeric bio-adsorbent composed of cellulose-g-poly-(acrylamide-co-sulfonic acid) (CASA) prepared by grafting copolymerization is an interesting candidate for a sorbent that can be applied for Cr(III) adsorption from leather wastewater [57]. SEM, XRD, FTIR, and XPS results showed that CASA contained many spherical particles and functional groups such as -NH_2_, -C=O, and -HSO_3_, and due to this, the material was an excellent sorbent of Cr(III) (274.69 mg/g of max adsorption capacity) from high-salinity wastewater. In addition, the adsorption process followed the Langmuir adsorption isotherm, and the experimental data conformed to the pseudo-second-order kinetics model. The adsorption of Cr(III) on CASA proceeded according to chelation, electrostatic interactions, and cation exchange.

In addition, functional biobased hydrogels show superior adsorption performance for cationic contaminants via electrostatic and hydrogen (H)-bonding interactions [58]. A promising sorbent has been reported to be a biobased egg albumin (ALB) hydrogel functionalized with a large density of adsorptive amine sites via polyethyleneimine (PEI) [59]. For example, Godiya et al. [60] have presented a novel eco-friendly bilayer-amine group incorporated microcrystalline cellulose (MCC)/chitosan (CS) hydrogel, fabricated by integrating polydopamine (PDA) and polyethyleneimine (PEI) for reliable and effective extraction of heavy metal ions from effluents. Due to abundant adsorptive sites, the MCC-PDA-PEI/CS-PDA-PEI hydrogel showed excellent Cu^2+^, Zn^2+^, and Ni^2+^ adsorption capability values of 434.8, 277.7, and 261.8 mg/g, respectively. In a multi-ion adsorption system, the hydrogel removed mixed metal cations with slightly higher selectivity for Cu^2+^. A plausible binding mechanism of metal cations on the as-prepared hydrogel was indicated to run according to chelation between hydrogel functional groups and metal ions. In the repetitive adsorption/desorption experiments, the hydrogel retained an above 40% metal ion adsorption and desorption capacities after four cycles. Furthermore, the Cu^2+^-adsorbing hydrogel could serve as a support for the in situ development of Cu nanoparticles, which showed excellent catalytic performance, as demonstrated by the transformation of 4-nitrophenol to 4-aminophenol.

It is worth mentioning that desorption research is also being conducted, but less attention has been paid to recycling the used adsorbents and recovery of heavy metals from the desorbing agents. For regeneration and reuse of adsorbents, various possible regenerating agents such as acids, alkalis and chelating agents (such as ethylene diamine tetraacetic acid) have been proposed by many researchers with very limited success, and only up to a limited number of adsorption–desorption cycles. Only a few of the reported studies focused on the recovery of adsorbed (from saturated adsorbents) and desorbed metals (from regenerating agents). For example, Azam et al. [61] prepared low-cost adsorbents including raw date pits and chemically treated date pits, and applied these materials to investigate the adsorption–desorption behavior of Cr(III) and Cd(II) from wastewater. The maximum metal elution from these biomaterials (97%) was reached with 0.1 M HCl, while the elution of both Cr(III) and Cd(II) with various eluents increased in the following order: 0.1 mol/dm^3^ NaOH < 0.1 mol/dm^3^ CH_3_COOH < 0.1 mol/dm^3^ H_2_SO_4_ < 0.1 mol/dm^3^ HNO_3_ < 0.1 mol/dm^3^ HCl. An approximately 80% drop in Cr(III) and Cd(II) adsorption was observed after the five regeneration cycles of adsorption–desorption. Hence, the results demonstrated that the prepared materials could be a low-cost and eco-friendly choice for the remediation of Cr(III) and Cd(II) contaminants from aqueous solutions.

Cr(III) adsorption by chitosan, an effective and low-cost sorbent, in batch tests was carried out with a 76% recovery. The effective desorption of Cr(III) was then carried out simply by washing the used chitosan with 0.1 M EDTA solution due to formation of strong chelating Cr(III)-EDTA complexes. However, the use of EDTA is problematic because of low biodegradability of this chelating agent.

Different eluting solution was used after removal of Cr(III) from aqueous solution using *Bacillus subtilis* biomass [62]. The optimum pH and temperature for biosorption were found to be 4.0 and 60 °C, respectively. A biosorbent dosage of 1 g/dm^3^ showed maximum metal uptake (q_e_) of 23.9 mg/g for an initial metal concentration of 100 ppm. In the case of this biomass, Cr(III) was efficiently eluted (81%) with 5 M NaOH solution.

### 3.3. Ion Exchange

Ion exchange (IX) is a physical–chemical process that selectively removes contaminants from a solution by effectively swapping out ions of similar electrical charges. The material of solid ion-exchange particles could be either natural, e.g., inorganic zeolites, or synthetically produced, e.g., organic resins. Both organic and inorganic ion-exchange materials have limitations. Organic ion-exchange resins are poorly thermally stable. For example, the mechanical strength and heavy metal removal ability of ordinary organic ion-exchange resins tend to be lower at high temperature (e.g., for treating liquid radioactive waste) than at low temperature. Inorganic ion-exchange materials are poorly reusable, have low mechanical strength, and are not resistant to some chemicals [63,64]. The ion-exchange method can remove target ions of heavy metals (some or all), such as Cr(III) from wastewater [39]. The main drawback of the IX method is that it cannot be used to decontaminate the effluents containing high concentrations of metal ions because of the risk of destruction of the resin matrix. Moreover, IX is nonspecific because different factors such as pH, the presence of various anions, temperature, initial concentration of the resin and sorbate, and time of contact affect the ion-exchange operation [37]. Currently, inorganic–organic composite ion-exchange materials have been developing to overcome these limitations of organic ion-exchange resins and inorganic adsorbents. Composite ion-exchange materials combine the mechanical properties of organic polymers and the inherent properties of inorganic compounds, i.e., they have advantageous mechanical properties, are chemically inert, are stable at high temperature and when exposed to radiation, and can reproducibly and selectively remove heavy metal ions from solutions [65,66].

The applicability of natural exchangers such as clinoptilolite and synthetic zeolites to the removal of heavy metals from industrial effluents has been investigated. A synthetic zeolite has been reported to show a tenfold higher sorption capacity than that of clinoptilolite even having an almost similar surface area (20–28 m^2^/g). This may be attributed to the strength of hydration of the shell cations. Moreover, various researchers have also used other synthetic resins such as Amberlite-120 and Dowex 2-X4 to investigate the removal of heavy metal ions from wastewater containing Cr(III) and Cr(VI) ions [67,68].

It is also worth mentioning that ion-exchange resins based on water-insoluble polymers poly(acrylamide-co-styrene sodium sulfonate) (P(AAm-co-ESS)), poly(2-acrylamide-2-methyl-1-propanesulfonic acid-co-acrylicacid) (P(APSA-co-AAc)), poly(2-acrylamidoglycolic acid-co-2-acrylamide-2-methyl-1-propane sulfonic acid) (P(AAGA-co-APSA)), and poly(2-acrylamidoglycolic acid-co-4-styrene sodium sulfonate) (P(AAGA-co-ESS)) were synthesized by radical polymerization and were used to remove Cr(III) from an aqueous solution [69]. The synthesized ion-exchange resins exhibited excellent removal of Cr(III), i.e., 89.4, 88.3, 86.8, and 89.3% Cr(III) adsorption was reached with P(AAGA-co-APSA), P(AAGA-co-ESS), P(AAm-co-ESS), and P(APSA-co-AAc) resins, respectively. A breakthrough capacities of 1.5 and 1.2 mg Cr(III)/cm^3^ of the P(AAGA-co-APSA) resin were obtained in the first and second cycles, respectively. The elution efficiencies were 100 and 90.2% in each consecutive cycle of column adsorption.

### 3.4. Conventional and Unconventional Extraction

Liquid–liquid extraction (LLE), also called solvent extraction, is a mature conventional process that is applied for the separation of metal ions from various liquid solutions [70]. The crucial aspect for the efficiency and selectivity of LLE is the selection of an appropriate extractant from a wide selection of organic compounds classified as acidic (chelating and non-chelating), basic, or neutral (solvating) extractants [70,71]. The choice of extractant depends on the metal species existing in the spent liquor; cationic species are extracted by acidic extractants, whereas anionic species are removed by the basic ones. Cr(III) has been reported to be present in the form of Cr^3+^ at pH of less than 3.8 and change with increasing pH from Cr(OH)^2+^ (pH 4–6), through Cr(OH)_3_ (pH 7–11), to finally above pH 12, Cr(OH)_4_^−^ [1,13,20,72]. Furthermore, when LLE is applied for the removal of chromium from industrial effluents, the ageing of the solution must be taken into account. The phenomenon of ageing is related to the change in the type of Cr(III) species present in aqueous solutions, particularly in sulfates or chlorides, during storage time [73]. It means that the efficiency of Cr(III) extraction from fresh and aged (stored for more than a couple of hours) liquors can significantly differ, and designers of recovery processes must be aware of this issue. Another reason for a decrease in the efficiency of chromium ion extraction may be the formation of aggregates in the organic phase by basic or acidic extractants. Aggregates (e.g., dimers) behave as monomers, and extract only one monovalent anionic metal complex, thus reducing the performance of the organic phase, as a result of the reduction in the effective concentration of extractant [1,73].

For the last five years, conventional LLE of Cr(III) from the industrial effluents has not been widely investigated. However, in the first decade of the 21st century, interest in LLE use was observed. The extraction of Cr(III) with acidic or basic extractants from acidic media is pH dependent. Quaternary ammonium salts (basic extractants) were proposed to extract Cr(III) from tannery effluents previously diluted and sufficiently alkalized with NaOH to form Cr(OH)_4_^−^ anions (pH~13) [13,73,74]. Methyltrioctylammonium chloride (MTOACl, commercial name Aliquat 336) was proven to efficiently extract (more than 98.5%) tetrahydroxochromate(III) anions from alkalized solutions according to the reaction (the o and a subscripts stand for organic and aqueous phases, respectively) [13,20]:

(1) $${\mathrm{MTOACl}}_{\left(o\right)}+\mathrm{Cr}{\left(\mathrm{OH}\right)}_{4\left(a\right)}^{-}=\mathrm{MTOACr}{\left(\mathrm{OH}\right)}_{4\left(o\right)}+{\mathrm{Cl}}_{\left(a\right)}^{-}$$

Cr(III) loaded into the organic phase should be subsequently stripped to recover it to an aqueous phase and proceed to further treatment. Solutions of sulfuric acid (e.g., 0.5 mol/dm^3^) were proven to strip Cr(III) easily and with high yield. The enrichment ratios of Cr(III) for a one-step extraction–stripping process ranged from 1.0 to 8.4 depending on the A/O volume phase ratio [13].

Based on LLE, a hydrometallurgical separation of vanadium from chromium has been proposed [20]. An ilmenite paste was contacted for 2.5 h with 6.0 mol/dm^3^ H_2_SO_4_ in an acid-to-paste mass ratio of 9:1 to leach metal ions (composition of the leach liquor in Table 1). Depending on the pH of the aqueous phase, various metals can be selectively extracted. Thus, V(V) was separated from Cr(III) from ilmenite leach sulfate solutions by 0.4 mol/dm^3^ Aliquat 336 in kerosene at low pH values (0−2.0), 99.7% E_V(V)_. With increasing pH, selectivity decreased and Cr(III) was extracted in preference to V(V) at pH 9.0−12.0, 99.8% E_Cr(III)_. V(V) was efficiently stripped (99.9%) from the organic phase with 8.0% NH_4_OH, while almost 100% Cr(III) was stripped with 0.7 mol/dm^3^ H_2_SO_4_. Finally, V(V) and Cr(III) were precipitated from the corresponding stripping solutions, separated and calcined at 500 °C, resulting in pure V_2_O_5_ (97.34% V_2_O_5_, 0.59% Cr_2_O_3_, 0.25% Al_2_O_3_, ≤1.82% of other impurities) and Cr_2_O_3_ (98.03% Cr_2_O_3_, 1.29% Al_2_O_3_, ≤0.68% of other impurities).

**Figure 3:**
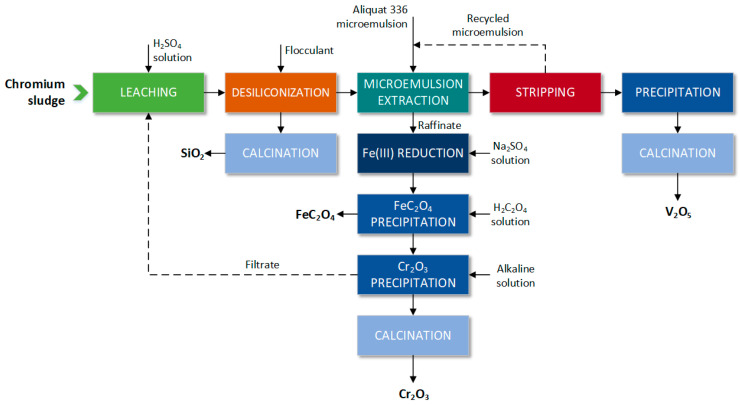
Scheme of the separation of V(V) from Cr(III) using an Aliquat 336 microemulsion (according to [21]).

The separation of vanadium from chromium has also been performed by an unconventional extraction using the Aliquat 336 microemulsion [21]. Although this multistep process appears to be complicated (Figure 3), its high efficiency and selectivity make it a potential alternative to sludge landfilling.

The leach solution (see Table 1) obtained after leaching of a chromium sludge with sulfuric acid was contacted with cationic polyacrylamide (CPAM, M_w_~8 mln) to remove SiO_2_ (93% desiliconization) and to prepare white carbon black (almost 95% pure). Subsequently, V(V) was selectively extracted from the solution after desiliconization, and separated from Cr(III) and other ions by microemulsion. The structure of Aliquat influences the surface active properties of the compound; therefore, it performs not only as an extractant but also as a cationic surfactant stabilizing the microemulsion. Since V(V), Cr(III) and Fe(III) existed in the leach solution as anionic H_2_V_10_O_28_^4−^ and cations of Cr^3+^ and Fe^3+^, Aliquat 336 selectively extracted more than 98% vanadium by anion exchange reaction:

(2) $$2{\mathrm{MTOACl}}_{\left(o\right)}+H_{2}V_{10}O_{28\left(a\right)}^{4-}+2H_{\left(a\right)}^{+}={\left(\mathrm{MTOA}\right)}_{2}{\left(H_{4}V_{10}O_{28}\right)}_{\left(o\right)}+2{\mathrm{Cl}}_{\left(a\right)}^{-}$$

Eventually, after subsequent stripping–precipitation–calcination, the final product V_2_O_5_ (99% purity) was obtained and the total recovery of V(V) was 95.5%, while Cr(III) cations remained in the leach solution and were precipitated as Cr(OH)_3_, further calcined to Cr_2_O_3_ (98% purity). The overall recovery of Cr(III) in this process was almost 93%. Furthermore, Fe(III) was reduced to Fe(II) and precipitated as FeC_2_O_4_ by oxalic acid. Moreover, the microemulsion and residual water were recycled and reused in the process, reducing the excessive consumption of these resources. In summary, it should be emphasized that the mass exchange area expanded by microdroplets of the extractant makes microemulsion extraction advantageous over the conventional one. As a result of the effective recovery of the main components of the chromium sludge and the recycling of water and microemulsion, the proposed process can be considered a low environmental impact technology.

*Bis*(2,4,4-trimethylpentyl)phosphinic acid (commercially called Cyanex 272), an acidic extractant, was proposed to separate Zn(II) and Fe ions from Cr(III)-containing passivation baths (composition of the bath in Table 1) [19]. Separation was reached with 10% *v*/*v* Cyanex 272 in Shellsol D70 diluent at O/A ratio 1. Nearly 90% Zn(II) and 100% Fe ions were extracted at the pH range of 2.5 to 3.5 according to the cation exchange reaction (HR and M^n+^ indicate the acidic extractant and metal ions, respectively):

(3) $${\mathrm{nHR}}_{\left(o\right)}+M_{\left(a\right)}^{n+}={\mathrm{MR}}_{n\left(o\right)}+{\mathrm{nH}}_{\left(a\right)}^{+}$$

In acidic medium, Cr(III) was not extracted by Cyanex 272 (less than 7%) and remained in the raffinate. Zn(II) and Fe ions were recovered from the loaded organic phase by stripping with 2 mol/dm^3^ H_2_SO_4_.

**Figure 4:**
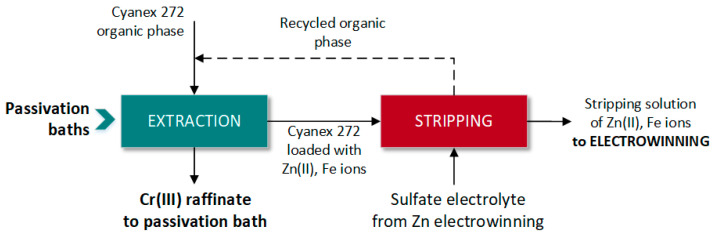
Extraction–stripping process for purification of Cr(III)-containing passivation baths (according to [19]).

Based on the results obtained, a hydrometallurgical process including one extraction step and two stripping steps was proposed to purify the spent passivation baths (Figure 4). The stripping solution containing Zn(II) and Fe ions could be processed by electrowinning to deposit metallic zinc (iron presence was stated not to disturb zinc deposition), and the depleted electrolyte was proposed to be used as a stripping phase.

In addition, emulsion pertraction technology (EPT) was proposed for the separation of Zn(II) and Fe ions from Cr(III) by extraction [22,23]. EPT is another technical solution of the extraction–stripping process using the hollow fiber membrane module (HF), combining in one HF module the extraction of metal ions to an organic phase containing an extractant, and the stripping to a receiving phase dispersed in the organic phase, forming a pseudo-emulsion. Although supported liquid membranes are used in this process, the core of the Cr(III) separation is extraction and stripping; therefore, EPT is presented in Section 3.4, and not in Section 3.5. It was shown that EPT using Cyanex 272 was successfully applied for the continuous purification of Cr(III) passivation baths on the laboratory and industrial scale from Zn(II) and Fe impurities. The advantages of EPT over the conventional extraction are easy scaling-up of the technique, large mass exchange area of the HF module, easy integration of EPT in the continuous passivation operation, flexibility of the design, reliability, and compactness of the process.

An acidic extractant 0.1 mol/dm^3^ Cyanex 301 (*bis*(2,4,4-trimethylpentyl)dithiophosphinic acid) in toluene was shown to selectively extract only impurities that accompany Cr(III) ions in plating baths such as Fe(III), Co(II), Ni(II) and Cu(II) [75]. The plating bath was alkalized with MgO to precipitate Cr(OH)_3_ and impurities, and afterwards the deposit was dissolved in 6 mol/cm^3^ H_2_SO_4_. This pretreatment step was necessary to preconcentrate the metal ions. The impurities (Fe(III), Co(II), Ni(II), Cu(II)) were shown to be extracted almost quantitatively from 0.1 mol/dm^3^ HCl or H_2_SO_4_ and the extraction effectiveness decreased with increasing acid concentration. In conclusion, LLE with Cyanex 301 was successfully used to purify Cr(III)-rich solutions (2.6 g/dm^3^ Cr(III)) for the preparation of plating baths and also to reuse it and, as a result, to reduce disposal/loss of chromium.

**Figure 5:**
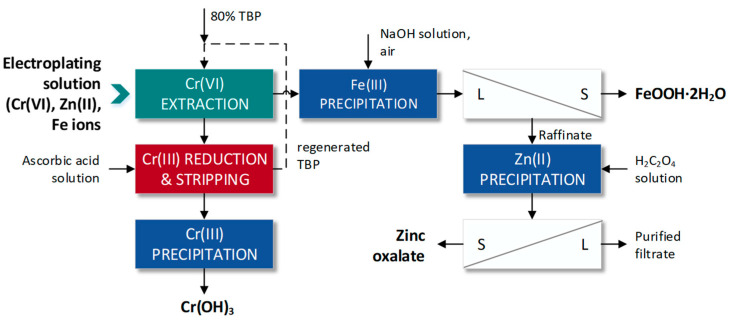
Scheme of removal of Cr(VI) by LLE and reduction to Cr(III) (according to [18]).

Another approach to the removal of chromium from an electroplating solution containing Cr(VI) by extraction has been presented by Ishfaq et al. [18] (Figure 5).

In this research, LLE with 80% tributylphosphate in kerosene (TBP, solvating extractant) was performed to remove Cr(VI) from the feed solution. To extract Cr(VI) efficiently, at least three extraction stages were necessary at O/A volume ratio 1. Subsequently, the loaded TBP phase was contacted with a solution of 2 mol/dm^3^ ascorbic acid to reduce Cr(VI) to Cr(III) and also to strip chromium ions (99% efficiency) from the organic phase (Equation (4)).

(4) $$2{\left[{\mathrm{HCrO}}_{3}\mathrm{Cl}\cdot 2\mathrm{TBP}\right]}_{\left(o\right)}+3{\left[C_{6}H_{8}O_{6}\right]}_{\left(a\right)}=2{\mathrm{Cr}}_{\left(a\right)}^{3+}+4{\left[\mathrm{TBP}\right]}_{\left(o\right)}+3{\left[C_{6}H_{6}O_{6}\right]}_{\left(a\right)}+2{\mathrm{HCl}}_{\left(a\right)}+6{\mathrm{OH}}_{\left(a\right)}^{-}$$

Then, Cr(OH)_3_ was precipitated from the stripping solution at pH 9.0, and FeOOH·2H_2_O deposit was obtained from the raffinate at pH 3.5. Finally, zinc oxalate was precipitated with 99.8% yield from Fe-free raffinate. Thus, the low-environmental impact of the proposed process was shown because it led not only to the removal of toxic or heavy metals but also to the recovery of chromium in the less toxic form of Cr(III) and zinc oxalate as a product.

Generally, it is visible that in most cases of waste effluents presented by the researchers, extraction has been used to remove various metallic impurities and to purify Cr(III)-containing solutions before reusing these solutions.

### 3.5. Membrane Techniques

Membrane techniques are becoming increasingly important in industrial applications, allowing, among other solutions, efficient treatment of wastewater and recovery of valuable raw materials [76,77]. Examples of such applications in the treatment of wastewater containing Cr(III) ions include single membrane techniques and hybrid systems, which combine several methods to improve process efficiency. It should be noted that this trend of combining several well-known methods into a comprehensive solution has received considerable attention. An example of such an approach to the wastewater problem is the solution proposed by Selvaraj et al. [24] for the use of electroflotation with a Nafion 117 membrane to recover Cr(III) from wastewater in a tannery and its reuse in the cowhide leather tanning process. This solution allowed 98% of the chromium to be recovered. Due to the use of the membrane, there was no need to oxidize chromium to hexavalent, which not only reduces the number of operations performed, but also allows chromium to be easily recycled into the process and eliminates the problem of a much more toxic form of chromium appearing in the system. Solutions in which several membrane modules are connected in a series are also used, for example, as in the approach proposed in [26], i.e., the combination of ultrafiltration (UF) and reverse osmosis (RO) modules. This method allows the potential of both membrane filtration techniques to be exploited. On the one hand, by using an ultrafiltration module, it is possible to separate macromolecular compounds from wastewater at relatively low transmembrane pressures, which consequently protects the RO membrane by reducing the risk of fouling. On the other hand, by using an RO membrane, it is possible to separate metal ions, such as Cr(III).

**Figure 6:**
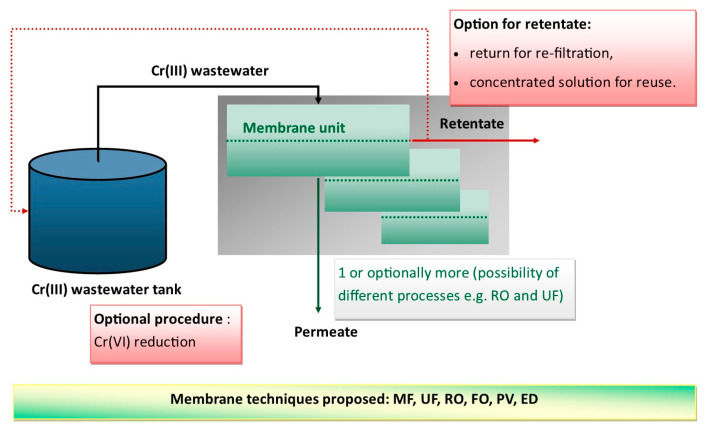
Schematic illustration of membrane processes for the removal of Cr(III) from wastewater.

It is worth noting that in the case of membrane techniques, most of the work is based on model solutions. Only a few papers have concerned the testing of real wastewater. This is a serious limitation in assessing the feasibility of using such solutions for industrial purposes. Recent works are summarized in Table 5, while a schematic illustration of the membrane process is presented in Figure 6. The lack of such reports may be due to a number of factors, including the difficulty of obtaining such an effluent, as well as the possible risk of not achieving very good results due to a number of additional foreign substances in the separated solution. Of course, it should be noted that modeling studies are often necessary for the development of optimal industrial processes and, therefore, cannot be neglected. For example, article [78] demonstrated the possibility of efficient co-removal of three- and hexavalent chromium by a positively charged UiO-66-NH_2_ decorated composite ultrafiltration membrane (polyethylene imine (PEI) based on polyvinylidene fluoride (PVDF). The authors are convinced that the new type of membrane proposed will be an excellent solution for real industrial wastewater. In support of this, several tests have been carried out in the presence of co-ions: Na^+^, Mg^2+^, Cl^−^, NO_3_^−^ and SO_4_^2−^. Moreover, effective regeneration with water or 0.1 mol/dm^3^ HCl was confirmed. Another research issue addressed was also the evaluation of the feasibility of Cr(III) removal by prereduction in Cr(VI) by chemical and photocatalytic reduction [79,80]. In this case, for example, a catalytic chemical reduction system in the presence of oxalate and membrane filtration was proposed [81]. Fe(III) and Al(III) ions added to the system result in the formation of Fe(III)-Cr(III) or Al(III)-Cr(III) agglomerates, which can be efficiently removed by a microfiltration (MF) membrane. In this case, the authors also see a high potential for the industrial application of such a process. An interesting solution is the use of liquid emulsion membranes for both the reduction of Cr(VI) to Cr(III) and its removal [82]. Studies have been conducted for real industrial wastewater from electroplating, indicating the feasibility of such a system. The use of liquid membranes in the removal of Cr(VI) ions has also been demonstrated in real wastewater. The authors in the article [83] successfully used the liquid emulsion membrane (ELM) to remove 97% of CrO_4_^−^ and Cr_2_O_7_^2−^ ions from the real rinse electroplating wastewater. For this purpose, a membrane composed of methyltrioctylammonium chloride, palm oil, and NaOH as a carrier, diluent, and stripping agent was applied. However, it should be noted that at the moment, liquid membranes are still in the stage of laboratory research, mainly because of the difficulty in maintaining the stability of such a system. Such an example of working in model systems is the synergistic extraction of Cr(III) by a supported liquid membrane (SLM) with the organophosphorus acidic extractants D2EHPA and Cyanex 272 [84]. Among membrane techniques, electrodialysis (ED) is also proposed, but mainly not for real industrial effluents [85,86,87].

### 3.6. Microbial-Based Techniques

Biological processes that can be proposed as an alternative for the treatment of Cr(III)-containing effluents from the tanning industry include the following aerobic or anaerobic processes [27,90]: (a) biotransformation conducted through the interaction arising between metal, proteins, and enzymes present in the cell membrane (reduction of Cr(VI) into simpler forms, less toxic and mobile); (b) bioadsorption/biosorption independent of the cell metabolism, consisting of a bond formed among metal and functional groups (e.g., carboxyl, sulfonate, amide, amine) in the cell wall, often on non-living biomass, and the use of traditional bioadsorbents as organic waste, algae biomass, fungi and yeasts; (c) bioaccumulation on living biomass, which is an intracellular process depending on the metabolism and the energy required to transport the metal across the cell. In anaerobic conditions, smaller pollution, greater selectivity, low cost, and tolerance to high concentrations of this toxic metal (e.g., 2760 mg of Cr(III)/dm^3^) are indicated as the process advantages. The Cr(III) removal under aerobic conditions has been carried out with microorganisms isolated from sludges or wastes containing ions of this metal.

Generally, Cr(VI) is reported to easily cross biological membranes of living organisms and to be reduced to Cr(III) inside the cells. On the contrary, the ways Cr(III) is transported into cells are poorly understood [91]. Some examples of microorganisms applied for the removal of Cr(III) from industrial effluents are given in Table 6.

*Penicillium* sp. isolated from a tannery effluent efficiently removed even an 84% Cr(III) from the wastewater at 35 °C and pH 4. However, it was reported that in spite of the endothermic effect of the sorption, the increase in temperature to 40 °C negatively influenced the stability of the fungal structure resulting in a decrease in Cr(III) biosorption [92].

The use of green alga *Chlamydomonas reinhardtii* as well as Cr(III)-tolerant *Dictyosphaerium chlorelloides* or *Spirulina platensis* as biosorbents for the removal of Cr(III) and Cr(VI) from model or real wastewaters has been reported [91,96,97]. Adsorption with alga cells is an example of bioaccumulation in the cytoplasm, vacuoles and chloroplast, and bioadsorption (subcellular partitioning in cell walls). Both forms of chromium have been proven to accumulate in a similar manner in cells. At the beginning of chromium removal, extracellular adsorption was observed, while after a longer time (72 h) Cr ions were adsorbed in intracellular matter [91]. However, the similar behavior of Cr(III) and Cr(VI) to subcellular components in algal cells suggested the intracellular binding of Cr by the same ligands, and could be explained by the rapid reduction in Cr(VI) just after it was transported inside the cells. The algae biosorbents bind Cr(III) due to the functional groups such as carboxyl, hydroxyl, amino, and phosphate groups on the surface of algae.

*Bacillus subtilis* VITSCCr01, isolated from the tannery polluted environment of the Palar river basin in India, showed tolerance of up to 1500 mg Cr(III)/dm^3^ and the Cr(III) bioremoval capacity of 64% [94]. Thus, these bacteria could be used to remove Cr(III) from both the environment and the tannery effluents.

*Escherichia coli* with cell-surface display of the cysteine-rich protein MerP (strain M-BL21) immobilized on magnetic pellets was reported to remove more than a 90% Cr(III) from a model solution, and 88% from real tannery wastewater compared to magnetic carrier alone (67%) [93]. The presence of microorganisms on the surface of the pellets enhanced the removal of Cr(III) because a cysteine-rich protein (MerP) contains two amino acids separated by cysteine residues that can form sites for the binding of Cr(III). It should be emphasized that the derivatized magnetic pellets are reusable and retain their magnetic properties after the bacteria immobilization and Cr(III) sorption.

A combined chemical–biological treatment system was proposed for Cr removal and recycling, i.e., chemical precipitation of a 98% Cr(III) with lime and cement dust, and simultaneous biological removal of a 99% Cr(VI) with actinomycete strain *Kitasatosporia* sp. from tannery wastewater [29]. After removal from the wastewater, Cr(III) was recovered from the precipitation and recycled to the leather tanning solutions, showing similar properties of the experimental leathers as the leathers tanned with commercial Cr(III) solutions. Thus, the proposed precipitation–biosorption process was concluded to be a promising approach to the treatment of tanning effluents from an economic and environmental point of view.

A native microalgae consortium (NMC) isolated from a wastewater treatment plant containing *Tetradesmus* sp., *Scenedesmus* sp. and *Ascomycota* sp. was used to remove Cr(III) from the real tannery effluent [28]. The NMC was enriched in a photobioreactor. The NMC presented the potential to obtain biofuels. The adsorption efficiency of microalgae was indicated to reach 99% of 100 mg/dm^3^ Cr(III). Thus, the efficient biosorption of Cr(III) made NMC a promising by-product from a future sustainable biorefinery, achieving a resource-efficient biomass use for a circular bioeconomy.

Finally, the biotransformation of Cr(VI) to Cr(III) was reported to occur in Cr-resistant bacteria (both aerobic and anaerobic microorganisms, *Pseudomonas aeruginosa* or *Mammaliicoccus sciuri*), and was catalyzed by chromate reductases, mediating electron transfer from electron donors to Cr(VI) [98].

### 3.7. Electrochemical Techniques

Wastewater containing chromium compounds is characterized by the presence of Cr(III) and Cr(VI) ions. In view of the toxicity of chromium species (especially Cr(VI)), limiting Cr(III) pollution also seems reasonable. The scientific literature indicates practically one method for Cr(III) removal from wastewater by electrochemical techniques. It involves dissolving anodes with a metal other than chromium and then precipitating Cr(III) compounds [99,100,101]. Cr(III) reduction studies were based on solutions from tanneries (real or model). Cr(III) used in the tanning process is not consumed completely. Approximately 30–40% of it is released into the environment. The process of Electro-Chemical Peroxidation (ECP) process was investigated [100]. In the first step, iron anodes were electrochemically dissolved to obtain Fe(II) cations. Then, the Fe^2+^ cations were oxidized by H_2_O_2_ to Fe^3+^ ions, followed by Cr(III) coprecipitation as Cr(OH)_3_ with Fe(OH)_3_. Other processes are also likely, such as reactions of Cr(VI) traces with Fe^2+^ (cations before oxidizing with H_2_O_2_ or after reduction from Fe^3+^ on cathode) and reduction to Cr(III). The optimum values of the ECP parameters for the process were found to be pH 2, 2 mg/dm^3^ Fe^2+^, 15 mg/dm^3^ H_2_O_2_ and a current density of 30 mA/cm^2^. Under these conditions, the Cr(III) concentration was reduced from 16 to 2 mg/dm^3^. The authors concluded that the electrochemical peroxidation process proved to be an efficient and appropriate technique for the removal of Cr(III) from tannery wastewater. A carbon steel anode was also used by Bonola et al. [101]. The TiO_2_/RuO_2_ cathode was the preferred due to better process kinetics, especially at current densities below 20 mA/cm^2^. Above 30 mA/cm^2^, the kinetics of the process on the mixed oxide or 316L stainless steel cathode were similar. Chromite (FeCr_2_O_4_) and small amounts of Cr(OH)_3_, CrO(OH) were observed in the precipitate. It was necessary to increase the pH to a level of 5–6. At pH = 3.55, the co-precipitation did not occur. An analysis of the use of other materials for anodes (Zn and Mg) is planned in the future. A similar process of electrocoagulation was proposed with aluminum anodes [102]. In addition, electrocoagulation was combined with biosorption using eggshells. The optimum value of the current density was 20 mA/cm^2^. The authors concluded that it is a feasible and low-cost process. Ramirez et al. [99], on the other hand, used copper anodes for the electrochemical dissolution of the anodes to generate Cu^2+^ ions. As a consequence of the electrochemical reaction, hydrogen was released on the graphite cathodes. It generated the alkalization of the solution and precipitation of Cr(III) hydroxide. Recovery efficiency was above 99%.

## 4. Summary and Future Perspectives

Generally, most examples of chromium removal from industrial wastewaters are related to Cr(VI) removal or reduction to Cr(III). However, Cr(III) must also be removed from industrial solutions, to prevent a release of chromium to the aquatic environment and a threat of Cr(VI) generation as a consequence of Cr(III) oxidation. The advantages and disadvantages of the operations applied for Cr(III) from industrial effluents are summarized in Table 7.

The presented review shows that precipitation seems to be the simplest operation to remove Cr(III) from the effluents; however, it cannot be considered sustainable or environmentally friendly, although adsorption, especially with biomaterials–low-cost adsorbents or microbial biomass, seems to be the most commonly applied operation for effective removal of Cr(III) from wastewaters. Furthermore, the biobased hydrogels fabricated by introduction of oxygen, nitrogen and sulfur comprising groups and inorganic fillers (e.g., graphene, clay, carbon nanotubes) seem to be attractive sorbents because of their distinctive physicochemical properties, cost effectiveness, ease of fabrication and operation, large specific surface area and porosity, and easy reusability [58,59].

The examples of separation systems that involve adsorption or membrane techniques show great potential for use in industrial applications. For example, membrane techniques are considered Best Available Techniques (BAT) in a number of wastewater treatment processes. Although membrane techniques are still considered costly and problematic, mainly due to membrane fouling, it is expected that they will be increasingly used (see Table 7). This is mainly due to the fact that materials engineering is developing rapidly. Specialists can design a process-resistant membrane, and modify the surface of the membrane to minimize the fouling phenomenon. For the most widely used polymeric membranes, modification of the substrate (support layer), mainly interfacial polymerization, or addition of various substances during the polymerization process, e.g., nanoparticles (e.g., silica, Ag, Cu NPs), carbon nanotubes, carbon quantum dots, MOFs, and polymers (zwitterionic, biomimetic) have been proposed [77,103,104,105,106,107]. Moreover, optimal process conditions are still being developed, including pretreatment of the feed (chemical, mechanical, other separation techniques), development of membrane cleaning methodologies, or changing process conditions (pH, flow rate, etc.). Furthermore, smart solutions, including Artificial Neural Networks (ANN) and Machine Learning (ML), are increasingly being considered to improve the efficiency of membrane filtration, with the aim of controlling the process in real time and reacting quickly to any anomalies. It seems that this direction, not only in membrane techniques, but also in other unit operations, will become established in industrial applications, mainly due to better and more powerful computers that allow increasingly complex problems to be solved by artificial intelligence (AI) [108,109,110].

**Table 7:** Advantages and disadvantages of the Cr(III) removal techniques presented in the review.

Technique	Advantages	Disadvantages	Ref.
Precipitation	Simple design Low operating cost	Lack of chromium recycling Landfilling Secondary pollution by chromium ions	[1,18,111,112]
Adsorption/ion exchange	Simple design Low investment cost High adsorption capacity Broad availability of various adsorbents	Low efficiency Weak selectivity Large volumes of diluted eluents	[1,111,112]
Liquid–liquid extraction	Operational flexibility Broad selection of extractants High intensity of mass transport Mature conventional operation	Use of VOC diluents (fire hazard) Loss of the organic phase (solubility with water) Large volumes of A and O phases Problems with separation of the phases	[18,70]
Membrane techniques	Compact, modular construction Easy to combine with other techniques Easy scaling-up Large contact area	High operating cost Undesirable fouling, scaling, etc. Auxiliary operations required (cleaning, prefiltering)	[1,113,114]
Microbial-based	Sustainability of the bioprocess Low operating cost No need to separate biomass cultivation nor harvesting biomass from the environment	Limited by metal concentration tolerated by microorganisms Highly sensitive to operational conditions Necessity for external source of energy for cell growing	[27,90]
Electrochemical	High process efficiency Relatively low cost of the equipment	High operating cost due to high energy consumption	[100,101]

The selectivity of the operations used must be developed, especially if complex multi-component wastewaters are processed, to separate various valuable metals and use them as secondary resources instead of natural ores. Another challenge on an industrial scale is the processing of large volumes of the liquors and achieving the required final concentration level, high intensity and efficiency of the removal process.

All of the operations presented are rather easy to include in multistep processes for the treatment of industrial effluents, and can be used to remove Cr(III) successfully, efficiently, and, in some cases, selectively. They must also guarantee long-term stability of the operation under various process conditions and flexibility towards fluctuations of the effluent composition. Among the operations presented, bioprocesses are gaining increasing prominence in the field of effluent treatment, and they are considered as a low environmental impact technologies (showing low toxicity, high biodegradability) for the treatment of industrial solutions contaminated with heavy metals. According to the assumptions of the circular economy, efforts must focus on integrated processes involving biological operations based on waste biomass from biorefineries.

## Figures and Tables

**Table 1:** Origin and composition of Cr(III)-containing spent industrial effluents.

Origin	Composition	Ref.
Steel leaching	in g/dm^3^: 20.4–37.2 Ni(II); 11.4–21.4 Co(II); 13.4–24.5 Cr(III); 7.20–8.78 Al(III); 0.02–0.53 Cu(II); 0.04–0.138 Fe(III); 0.11 Na(I); 0.023 Mg(II); 0.023 Zn(II) in mol/dm^3^: 3.46–4.98 H^+^; 2.28–3.22 SO_4_^2−^; 0.16–0.34 Cl^−^	[16,17]
Ilmenite leaching	in mol/dm^3^: 2.1×10^−3^ V(V); 5.41×10^−3^ Cr(III); 0.627 Ti(IV); 0.39 Fe_total_; 2.73×10^−2^ Mg(II); 1.74×10^−2^ Al(III); 6.4×10^−4^ Ln(III); 6 H_2_SO_4_	[20]
Chromium sludge leaching	in g/dm^3^: 20.64 Cr(III); 2.87 V(V); 5.84 Fe(III); 2.01 Si(IV); 0.83 Ca(II); 0.70 Mn ions; 0.54 Mg(II) in H_2_SO_4_	[21]
Passivation bath	in g/dm^3^: 11–20.5 Zn(II); 3–7 Cr_tot_, in mg/dm^3^: 15–100 Fe_tot_; acidic pH	[19,22,23]
Spent tanning liquor 1	in mol/dm^3^: 0.042 Cr(III); 0.201 SO_4_^2−^; 0.35 Cl^−^; pH 4.35	[13]
Spent tanning liquor 2	in mol/dm^3^: 0.102 Cr(III); 0.324 SO_4_^2−^; 0.752 Cl^−^; pH 3.70	[13]
Tannery effluents (six different leather industries in Bara and Parsa districts (Nepal))	in mg/dm^3^: Cr 0.7–345	[10]
Tannery spent effluent collected from CSIR-CLRI (Central Leather Research Institute), Chennai	in mg/dm^3^: total Cr 2481; Cl^−^ 36,000; SO_4_^2−^ 28,480; protein 570; lipid 981; pH 4.4	[24]
Tannery wastewater after chemical treatment	in mg/dm^3^: total Cr 2007.08; Ca 755.3; Fe 1.998; Na 31,030; Ni 0.3054; Zn 20.69; SO_4_^2−^ 60,414.61; CN^−^ 2; pH 4.13	[25]
Tannery wastewater from Kombolcha Tannery Share Company, Ethiopia	in mg/dm^3^: total Cr 200; dissolved solid 3000; suspended solid 2100; pH 5.3	[26]
Tannery effluent from Mexico	in mg/dm^3^: 2760 Cr(III); 0.023 Cr(VI); 19,080 Na(I); 832.7 Ca(II); 0.14 Cu(II); 0.029 Pb ions; 0.014 Ni(II), pH 4	[27]
Tannery effluent from Mexico	in mg/dm^3^: 5061 Cr(III); 0.023 Cr(VI); pH 5.23	[28]
Tannery effluent from Old Cairo, Egypt	in mg/dm^3^: 2131 Cr(III); 821 Cr(VI); 249 SO_4_^2−^, pH 3.6	[29]
Chromite ore processing waste (Hackensack River (NJ, USA)	in mg/kg: Cr total 199–3970; Cr(VI) 0.3–19; As 8.9–59.6; Cd 0.7–9.6; Fe 11,100–47,500; Pb 44.7–281; Mn 232–585; Hg 0.08–2.45; Zn 95.3–597	[30]
Textile mill effluents (Eight textile industries in Delhi NCR, India)	in mg/dm^3^: Cr 0.11–0.21; Cu 0.17–0.28; Fe 0.39–0.90; Pb 0.02–0.10; Ni 0.11–0.22; Zn 0.11–0.51; Cd 0.01	[31]
Chrome plating industry wastewater	in mg/dm^3^: Cr(VI) 5721.95; Fe 79.5; Pb 1.095; Cu 28.3, pH 2.09	[32]
Steel industry slags	in mg/kg: Cr 2915; Zn 1084; Ba 380; Sr 266; Cu 175; Zr 109; V 92; Nb 62; Pb 59; Ni 26; Sn 15; Mo 11; Rb 11; As 10; Cd 8; U 4; Br 5; Ce, Co, La < 5; Y, Th, Bi, Ga < 3	[33]
Chromium slag from Chemical Holdings Co., Ltd. (Fuzhou, China) during chromium salt production	in mg/kg: Cr(III) 112; Cr(VI) 464; Ca 26,600; Mg 3160; Fe 4550; Al 64.9; Cd 1.3; Ni 3.2; Cu 5.8; Mn 10.2; As 4.6; Co 1.5	[34]

**Table 2:** Chromium removal efficiency at an optimum pH for the three precipitating agents.

Precipitating Agents	Optimal pH	Max% of Cr(III) Removal	Ref.
CaCO_3_	8.9	99.95	[42]
NaHCO_3_	8.3	99.97	[42]
MgO	8.9	99.98	[42]
NaOH	4–5	99.99	[17,43]
CaO	4–5	99.99	[17]
Ca(OH)_2_	>7	99.99	[43]

**Table 3:** A summary of the factors affecting the overall adsorption yield [46].

Factor Affecting Adsorption	Effect on Adsorption
pH	Hydrogen (H^+^) and hydroxide (OH^−^) ions react with the activated sites of the adsorbent depending on the pH of the effluent
pH at the potential of zero-point charge (pH_zpc_)	The point of zero charge (PZC) or zeta potential analysis of the adsorbents determine the surface charge of the adsorbent at various pH values and affords information for the attraction and repulsion. When the pH value is lower than that of the PZC, the acidic water donates more protons than hydroxide groups, and, therefore, the surface of the bioadsorbent becomes positively charged (attracting anions). On the contrary, the surface is negatively charged (attracting cations/repelling anions) when the pH value is above the PZC
Adsorbent dosage	An increase in the number of active adsorption sites positively affects the efficiency of the removal of contaminants or pollutants; however, a dose that is too high reduces the total uptake of pollutants
Temperature	Increasing temperature reduces the viscosity of liquors, which enhances the mobility of contaminants from the bulk solution to the surface of the adsorbent
Pressure	Intensifies the adsorption until the process reaches equilibrium
Surface area	Small particles have a larger surface area compared to the large particles of adsorbent, allowing greater adsorption to be achieved
Coexisting ions	Fewer types of ions coexisting in the effluent increase efficiency of adsorption

**Table 5:** Examples of membrane processes in Cr(III) removal.

Origin	Basic Process Parameters	Results	Ref.
Tannery industry	2-compartment membrane (Nafion 117) electroflotation reactor, Anode: RuO_2_/TiO_2_-Ti, Cathode: Ti, Catholyte: spent liquor effluent, Anolyte: 0.01 N H_2_SO_4_	Formation of an insoluble lipid–protein–Cr(OH)_3_ complex in the form of foam. The removal efficiency of Cr(III), lipid and protein = 98, 91 and 95%, respectively	[24]
	RO and UF membrane system (polymeric membranes AFC 99, AFC 30, FB 200, PCI membrane), pH 3.5–12; feed flow rate 0.36–0.72 m^3^/h; TMP 25–40 bar	Total Cr removal efficiency (both Cr(VI) and Cr(III)) up to 99.99%, optimal pH 6.6; flow rate 0.62 m^3^/h, TMP 40	[26]
Chromium slag during chromium salt production	Bipolar membrane electrodialysis (BMED) with H_2_O_2_ (oxidative conversion of Cr(III) to Cr(VI) in alkaline solutions, where OH^−^ form bipolar membrane)	Recovery of chromium up to 69%. During the purification process, chromium state conversion occurred, which contributed to its recovery	[34]
Rinse electroplating wastewater	Liquid membrane phase: palm oil as diluent, Span 80 as surfactant, methyltrioctylammonium chloride ([MTOA^+^][Cl^−^])) as an extractant; Strippant: 2.0 mol/dm^3^ thiourea in 2.0 mol/dm^3^ sulfuric acid	100% and 82% of Cr are extracted and then removed. Extraction to membrane phase: ${\left[{\mathrm{Cr}}_{2}O_{7}\right]}_{\left(a\right)}^{2-}+2{\mathrm{MTOACl}}_{\left(o\right)}\to {\left(\mathrm{MTOA}\right)}_{2}{\left({\mathrm{Cr}}_{2}O_{7}\right)}_{\left(o\right)}+2{\mathrm{Cl}}_{\left(a\right)}^{-}$ Reduction in Cr(VI) in the internal phase: ${\left(\mathrm{MTOA}\right)}_{2}{\left({\mathrm{Cr}}_{2}O_{7}\right)}_{\left(o\right)}+2\mathrm{HSC}{\left({\mathrm{NH}}_{2}\right)}_{2\;\left(a\right)}^{+}\to 2\mathrm{SC}{\left({\mathrm{NH}}_{2}\right)}_{2\;\left(a\right)}+2{\mathrm{Cr}}_{\left(a\right)}^{3+}$	[82]
Sewage wastewater	Pervaporation (PV) using polyvinyl alcohol (PVA)/sodium Y (NaY) zeolite membranes	The membrane allows for the selective separation of Cr(VI) and Cr(III). Cr(VI) was not detected in any permeates	[88]
Printing and dyeing factory	Forward osmosis (FO) with a TFC membrane, casting solution: 1.5 wt.% LiCl. Initial concentration in wastewater, in ppb total Cr 23.93, Sb 0.43, aniline 46.03	Rejection of Cr, Sb, and aniline, after 10 h of FO operation, 99, 98, 99.5%, respectively. Cr was mainly as Cr(VI)	[89]

**Table 6:** Microorganisms used for the removal of Cr(III) from industrial effluents.

Microorganisms	Remarks	Ref.
**Bioadsorption**		
*Penicillium* sp. (fungus)	84% Cr(III) sorption achieved at pH 4.0, 35 °C with 1% (*w*/*v*) biomass of <150 μm size from a model tannery effluent, in g/dm^3^: 0.319 CaCl_2_; 0.962 MgCl_2_·6H_2_O; 0.234 Na_2_S; 6.205 Na_2_SO_4_·10H_2_O; 1.119 NaCl; 200 ppm Cr(III)	[92]
*Escherichia coli* (bacteria) immobilized on magnetic pellets	2.38 mmol Cr/g cell, 88%, from a real tannery wastewater, in mg/dm^3^: 1580 Cr_total_, 1380 Cl^−^, pH 4.25	[93]
*Kitasatosporia* sp. (bacteria)	99% Cr(VI) sorption from a tannery effluent pretreated after previous Cr(III) precipitation (composition presented in Table 1)	[29]
**Bioaccumulation**		
*Bacillus subtilis* (bacteria)	Cr(III) from the tannery effluent in Vellore District (India) of various concentrations of metal ions (100 to 2000 mg/dm^3^), in 2760.023 Cr_total_, 2760 Cr(III)	[94]
A native microalgae consortium (NMC) isolated from a wastewater treatment plant, containing *Tetradesmus* sp., *Scenedesmus* sp. and *Ascomycota* sp. (microalgae)	99% Cr(III) sorption from a tannery effluent (composition presented in Table 1)	[28]
*Sargassum wightii* (microalgae)	88% Cr(III) sorption in 5 stages (2 ppm level achieved in the liquor), 35% after the first stage, from a real tannery solution of 750 ppm, pH 3.5–3.8	[95]

## Data Availability

Not applicable.
